# Artificial intelligence in the retraction spotlight: trends, causes and consequences of withdrawn AI literature through a systematic bibliometric review

**DOI:** 10.3389/frma.2025.1737168

**Published:** 2026-01-20

**Authors:** Kannan Sridharan, Gowri Sivaramakrishnan

**Affiliations:** 1 Department of Pharmacology & Therapeutics, College of Medicine & Health Sciences, Arabian Gulf University, Manama, Bahrain; 2 Bahrain Defence Force Royal Medical Services, Riffa, Bahrain

**Keywords:** AI, artificial intelligence, publication ethics, retractions, scientific misconduct

## Abstract

**Introduction:**

The rapid integration of artificial intelligence (AI) in scientific research has introduced new challenges to academic integrity, with increasing concerns about AI-related article retractions. This study conducts a comprehensive bibliometric analysis of retracted AI-related articles to characterize their prevalence, causes, and impact on scholarly communication.

**Methods:**

A systematic search was performed in Scopus using the terms “Artificial Intelligence” OR “AI” AND “retract^*^” without restrictions on publication year or language. Bibliometric parameters including publication timelines, journal metrics, citation counts, and retraction characteristics were analyzed using VOS Viewer, Bibliometrix, and SPSS. Statistical tests assessed correlations between key variables.

**Results:**

From an initial yield of 1,152 articles, 335 retracted publications met inclusion criteria after duplicate removal and screening. The analysis revealed that 46.3% (155/335) of retractions occurred in 2023, with a median retraction time of 550 days post-publication. Engineering accounted for 30.4% (102/335) of retractions, while 72.2% (243/335) originated from China. Compromised peer review was the most common retraction reason, though 37.9% (127/335) lacked specific justification. Strikingly, 51.1% (172/335) of retracted articles-maintained field citation ratios >1, indicating persistent scholarly influence. Articles in special issues showed significantly faster submission-to-acceptance timelines (*p* = 0.016). Journal editors initiated 98.5% (330/335) of retractions, while author responses revealed disagreement in 35.4% (34/96) of cases where feedback was available.

**Discussion:**

This study highlights systemic vulnerabilities in AI-related research publication, particularly concerning peer review integrity and prolonged retraction timelines. The continued citation of retracted articles underscores the need for improved retraction alert systems. These findings call for stronger ethical guidelines and technological safeguards to maintain trust in AI-driven scholarly outputs.

## Introduction

The advent of generative artificial intelligence (GAI), such as ChatGPT, has profoundly transformed scientific research and publishing. These tools offer considerable potential to enhance scholarly productivity by assisting with writing, literature searches, and democratizing access to scientific knowledge (Misra and Chandwar, 2023; Pereira et al., 2025). This is reflected in the exponential growth of AI-related publications, from the first article in 1960 to over 43,000 publications amassing nearly 357,000 citations (Anbalagan, 2024). In specialized fields like healthcare, publications surged from 527 in 2018 to 4,587 in 2023 (Senthil et al., 2024).

However, this rapid integration raises significant ethical and scientific concerns (Misra and Chandwar, 2023; Van Noorden and Perkel, 2023; Thirunavukarasu et al., 2023). A key issue is the questionable originality of AI-generated text and the fact that AI cannot meet authorship criteria requiring accountability for a manuscript's integrity (Fernando and Filippo, 2023; Mondal et al., 2025). In response, bodies like the International Committee of Medical Journal Editors (ICMJE) now mandate disclosure of AI tool use (Fernando and Filippo, 2023; Mondal et al., 2025). Failure to comply risks severe consequences, including a disturbing rise in AI-related retractions, particularly from certain regions and open-access journals (Lei et al., 2024). Enforcement remains inconsistent, with only 24% of top publishers having explicit GAI guidelines compared to 87% of top journals (Ganjavi et al., 2024).

Traditionally, retractions address severe violations like fabrication or plagiarism (Khan et al., 2024). AI-related retractions represent a novel threat where misuse, whether unintentional or deliberate, blurs the line between human oversight and machine-generated error, jeopardizing research credibility (Nwozor, 2025). Despite increasing reports, systematic analyses of these retractions remain scarce.

To address this gap, this study conducts a comprehensive bibliometric analysis of retracted AI-related articles. We aim to answer the following research questions:

- What are the scope, prevalence, and temporal trends of AI-related article retractions?
- What are the primary stated reasons and underlying patterns causing these retractions?
- What actionable insights can inform policies to mitigate risks and enhance transparency?

## Methods

### Search methodology

A bibliometric search was conducted in the Scopus database, one of the largest curated abstract and citation databases of peer-reviewed literature, to identify retracted articles related to AI. The search strategy employed the following query: TITLE-ABS-KEY (Artificial intelligence OR AI) AND (retract^*^). No restrictions were applied regarding language, publication year, or document type to ensure a comprehensive retrieval of relevant records. The final search was executed on April 10, 2025, to include the most recently retracted publications. As this study is primarily a bibliometric study, protocol was not registered.

### Eligibility criteria

The retrieved records were exported and screened for duplicates using Rayyan software™, a web-based tool designed for systematic review management. Two authors independently performed the deduplication process to minimize errors. Any discrepancy between the authors during screening were resolved through consensus-based discussion, ensuring uniformity in the selection process. The eligibility criteria were defined as follows. Inclusion criteria comprised: (1) articles that were formally retracted as per the Scopus database record; (2) articles whose primary focus involved AI, machine learning, deep learning, natural language processing, or related computational intelligence techniques, as determined by screening the title, abstract, and keywords; and (3) document types classified as original research articles, review articles, or correspondences (including letters, commentaries, and editorials). These document types were selected as they represent the primary vehicles for formal, citable scientific communication in journals, where retraction mechanisms are most consistently applied and reported. Exclusion criteria were: (1) non-retracted articles; (2) articles where AI was not a central component of the methodology or primary subject matter; (3) document types such as conference papers and book chapters. Conference papers were excluded to maintain a consistent analytical focus on the peer-reviewed journal literature, where retraction policies, notice formats, and indexing practices are more standardized, allowing for a more homogeneous and comparable dataset. Backward or forward citation tracking was not performed, as the study's primary aim was a direct analysis of retracted articles meeting the inclusion criteria, rather than an analysis of their citation network.

### Bibliometric analysis

For each retracted AI-related article, the following metadata and bibliometric indicators were extracted and analyzed:

- Article type: Classified as original research, review, or correspondence, based on the journal's designation.
- Publication context: Whether the article appeared in a regular issue or a special issue of the journal.
- Publication timelines: Key intervals were calculated using available dates: Submission to acceptance (peer-review duration), acceptance to publication (production speed), submission to publication (total processing time) and publication to retraction (time until retraction).
- Journal subject area: Categorized into engineering, education, therapeutics, diagnostics and others (encompassing interdisciplinary fields).
- Journal performance metrics: SCImago Journal Rank (SJR) quartiles (Q1–Q4) for the year of publication (Scimago Journal and Country Rank, n.d.). Journal impact factor, as reported on the journal's official website.
- Article impact metrics: Citation count (total citations post-retraction), altmetric score (measuring non-traditional impact, e.g., social media, policy mentions), field citation ratio from Dimensions AI (Dimensions AI, n.d.), which benchmarks an article's citations against similar publications in its field. A value >1 indicates above-average citation performance.
- Author and institutional analysis.
- Number of authors per retracted article.
- Affiliated institutions and countries (for first author and all co-authors).
- Ethical and funding disclosures.
- Funding declarations (presence/absence).
- Conflict of interest statements (reported or undisclosed).
- Retraction details: Stated reason for retraction (such as plagiarism, data fabrication, AI misuse).
- Retraction initiator: Authors (self-retraction), journal/editor (publisher-initiated), and third-party (e.g., institutional investigation).
- Author response to retraction: Agreed, disagreed, no response, uncontactable and not mentioned.
- Keyword analysis: Author-provided keywords were analyzed using multidimensional component factorial analysis to identify dominant themes and conceptual clusters.

The results were visualized using a range of graphical representations, including a PRISMA flow diagram, bar and box plot, cluster diagrams, a treemap, and thematic maps.

## Results

### Search results

**Figure 1:**
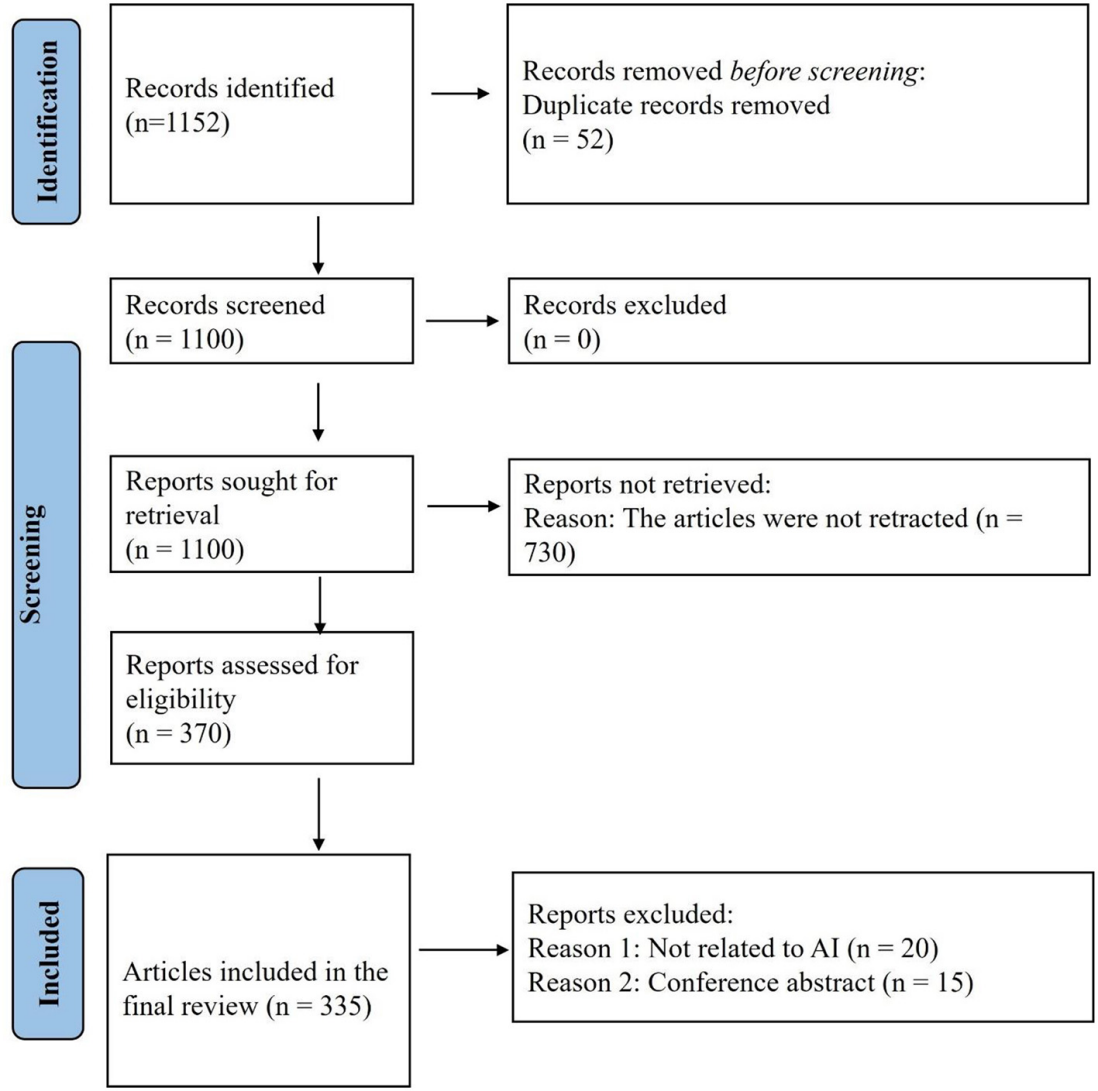
Study flow diagram. A total of 1,152 articles were obtained of which 335 were included in the final analysis.

The initial search strategy yielded 1,152 articles, from which 51 duplicates were excluded. Following further screening, 765 articles were removed for not meeting inclusion criteria, resulting in a final dataset of 335 retracted AI-related articles (Figure 1). Among these, the vast majority (325, 97%) were original research articles (one each was a systematic review and meta-analysis categorized as original research by the respective journals), while 5 (1.5%) were review articles and another 5 (1.5%) were correspondences. The publication context (regular vs. special issue) could not be determined for 3 articles; however, among the remaining 332, a substantial proportion (273, 82.2%) were published in special issues, with the rest appearing in regular issues.

### Timelines of the retracted AI-related articles

**Figure 2:**
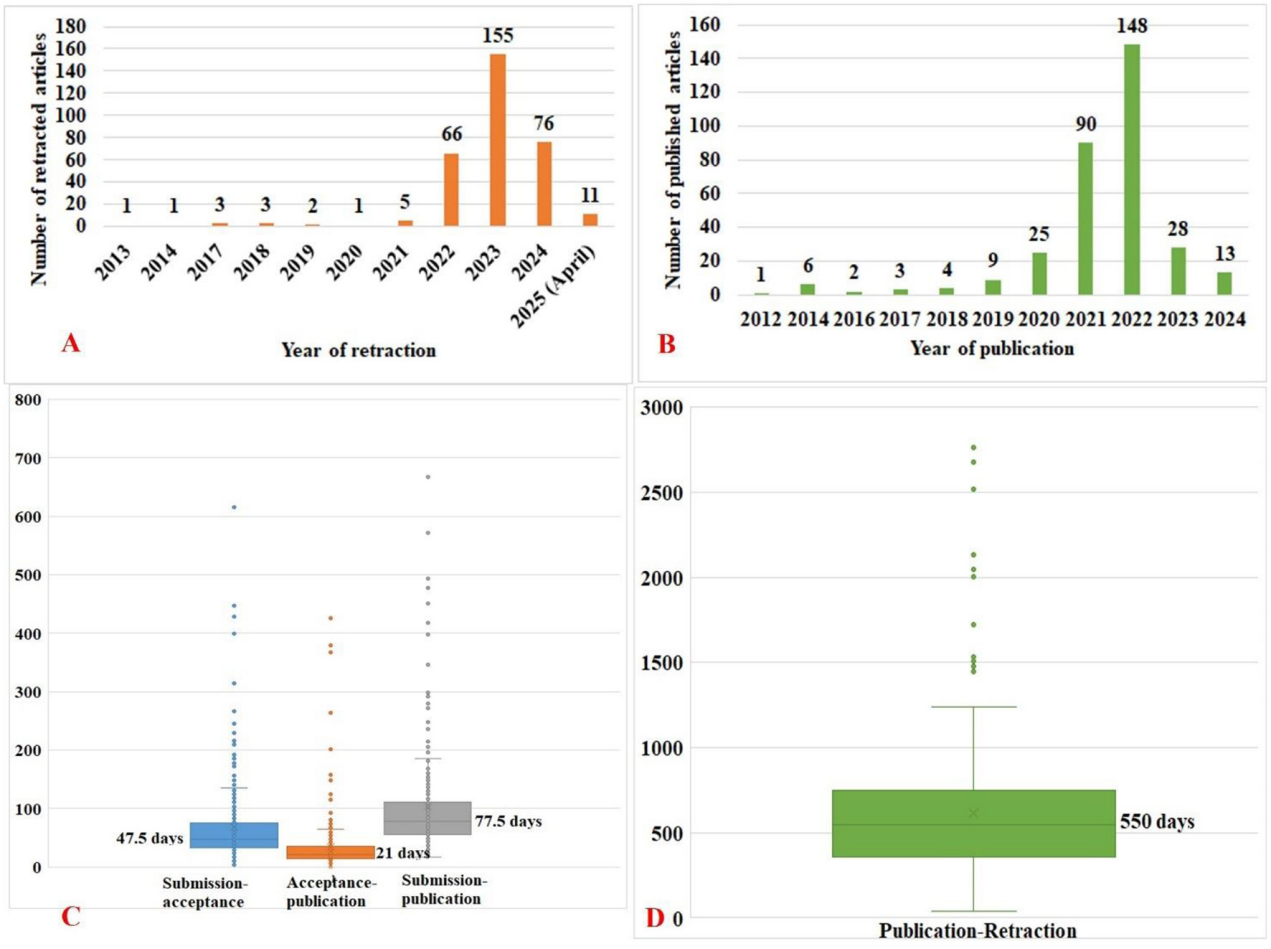
Timelines of the retracted AI-related articles. **(A)** Year-wise retractions of AI-related articles; **(B)** year-wise publications of AI-retracted articles; **(C)** timelines between various phases of publications; and **(D)** timeline between publication and retraction.

Retractions of AI-related articles spanned from 2013 to 2025, though the exact retraction date was unavailable for 11 articles. Strikingly, 155 (46.3%) of the 325 retractions with available dates occurred in 2023, followed by 76 (22.7%) in 2024 (Figure 2A). The original publication dates of these retracted articles ranged from 2012 to 2024, with the highest number (*n* = 148) published in 2022 (Figure 2B).

The median time intervals for key publication phases were as follows:

- Submission to acceptance: 47.5 days (range: 0–615)
- Acceptance to publication: 21 days (range: 0–426)
- Submission to publication: 77.5 days (range: 17–671; Figure 2C)
- Publication to retraction: 550 days (range: 41–2762; Figure 2D)

A statistically significant positive correlation (*r* = 0.34; 95% CI: 0.24–0.44; *p* < 0.01) was observed between the duration from submission to acceptance and the time until retraction.

**Figure 3:**
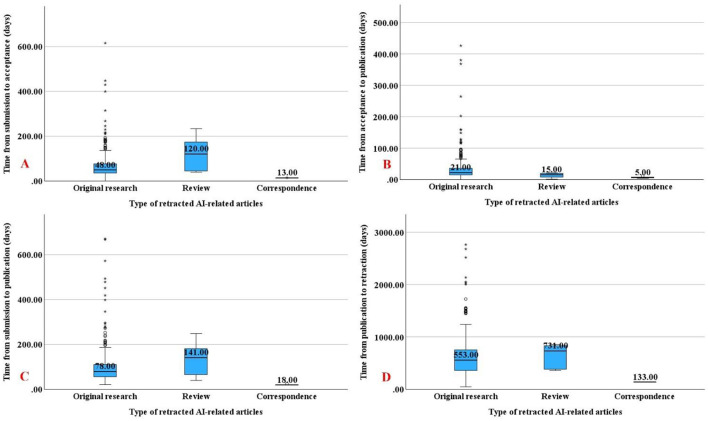
Timelines between publication phases and retraction according to article types. Box-plots depicting the timeframes from article submission until acceptance **(A)**, acceptance until publication **(B)**, submission until publication **(C)**, and publication until retraction **(D)**.

Correspondence articles exhibited significantly shorter timelines across all publication phases (submission to acceptance, acceptance to publication, submission to publication, and publication to retraction) compared to original research articles (*p* < 0.001; Figure 3). Similarly, correspondence articles had shorter intervals than review articles in all phases except acceptance to publication (Figure 3).

**Figure 4:**
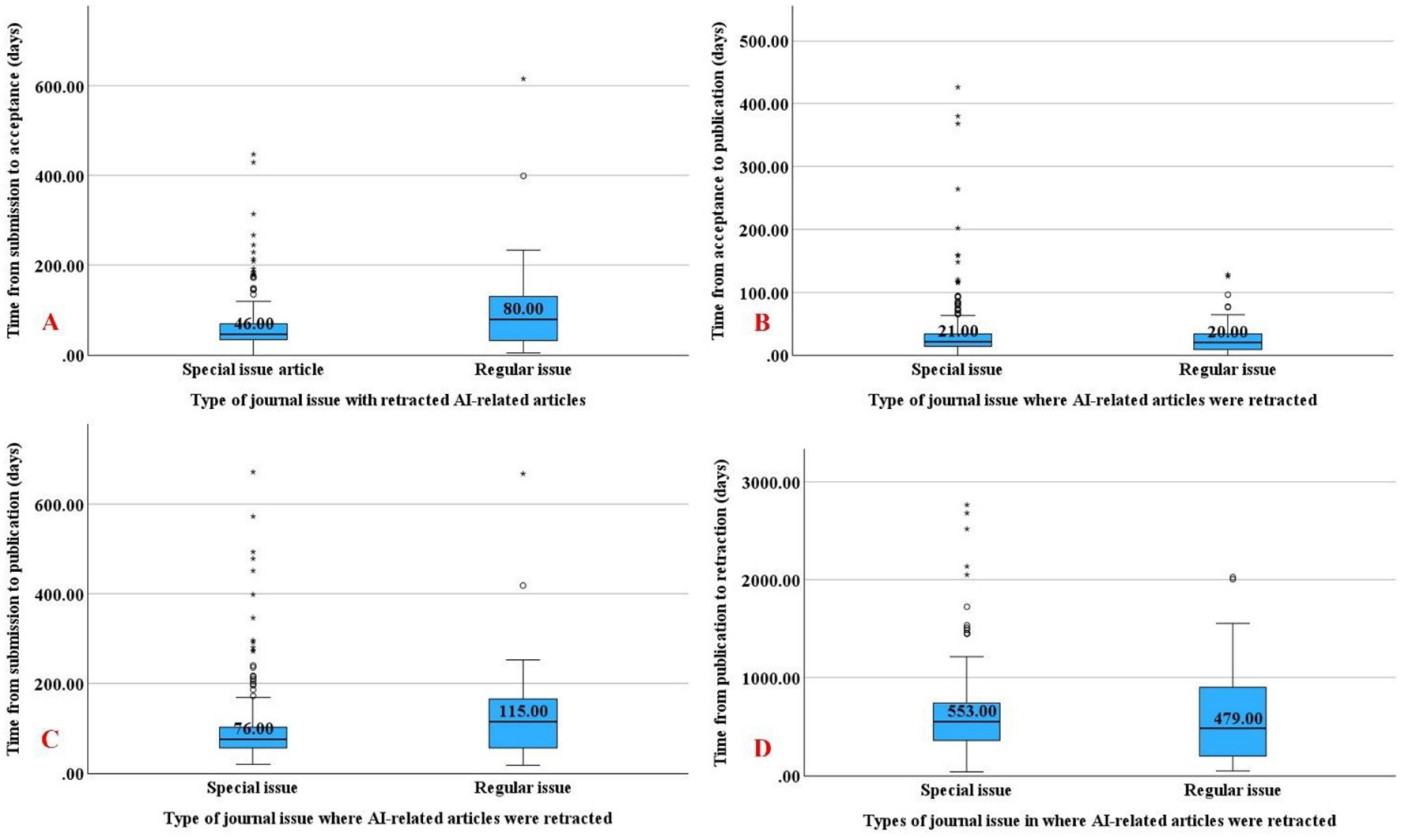
Timelines between publication phases and journal issues where AI-related articles were retracted. Box-plots depicting the timeframes from article submission until acceptance **(A)**, acceptance until publication **(B)**, submission until publication **(C)**, and publication until retraction **(D)**.

Further analysis revealed that retracted articles published in special issues had significantly shorter timelines between submission to acceptance (*p* = 0.016; Figure 4) and submission to publication (*p* = 0.025; Figure 4) compared to those in regular issues.

### Subject areas and journal performance indicators

The primary subject areas of journals publishing retracted AI-related articles were dominated by engineering (102, 30.4%), followed by education (41, 12.2%), therapeutics (35, 10.4%), diagnostics (15, 4.5%), and interdisciplinary categories (131, 39.1%). Among the top 10 journals with the highest number of retractions, nine specialized in engineering, while the remaining focused on agricultural and biological sciences (Supplementary Figure S1). The analysis of sources' local impact based on the H-index revealed that only a small subset of journals contributed substantially to the citation structure of the dataset (Supplementary Figure S2). The Journal of Ambient Intelligence and Humanized Computing showed the highest local impact (H-index of 4) followed by the Annals of Operations Research (H-index of 3).

Journal quartile analysis showed that most retractions occurred in Q2 (200, 59.7%), followed by Q1 (94, 28.1%), Q3 (40, 11.9%), and Q4 (1, 0.3%). Additionally, 146 (43.6%) of the retracted articles were published in journals that did not report an impact factor; among those that did, the median impact factor was 2 (range: 0.2–10.7).

### Impact of the retracted AI-related articles

**Figure 5:**
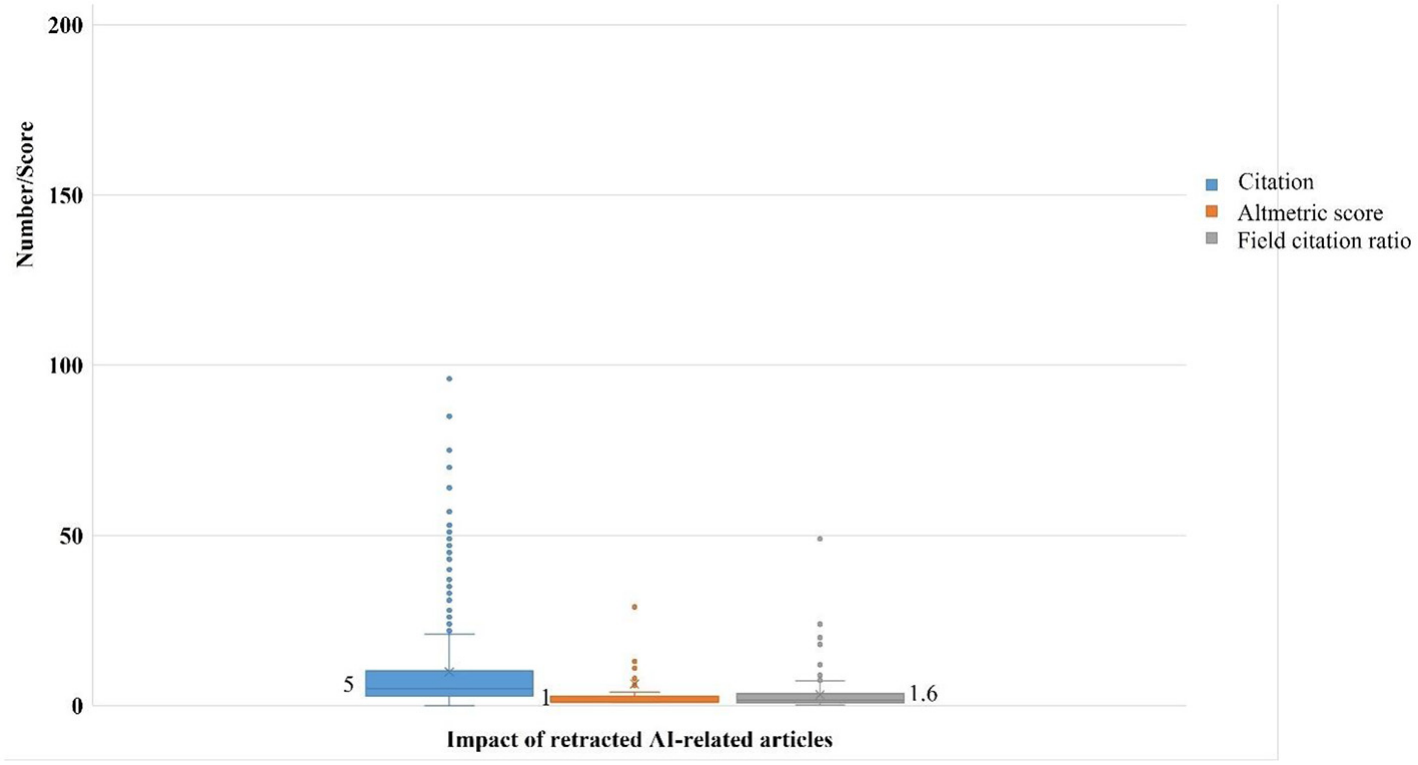
Impact measures of retracted AI-related articles.

The median citation count for retracted articles was 5 (range: 0–96), with an altmetric score of 1 (1–240) and a field citation ratio of 1.6 (0.2–49; Figure 5). Strikingly, 171 (51.1%) articles had a field citation report > 1, indicating above-average citation performance. Positive correlations were observed between the time from publication to retraction and both citation count (*r* = 0.4; 95% CI: 0.27–0.46; *p* < 0.001) and field citation ratio (*r* = 0.13; 95% CI: 0.04–0.25; *p* = 0.04), but not with altmetric score (*r* = −0.09; 95% CI: −0.32–0.19; *p* = 0.47).

### Authors, Institutions, and Countries

The median number of authors per retracted article was 2 (range: 1–11), with 89 (26.6%) being single-authored. Similarly, the median number of affiliated institutions was 1 (range: 1–11), and 171 (51.1%) listed only one institution.

**Figure 6:**
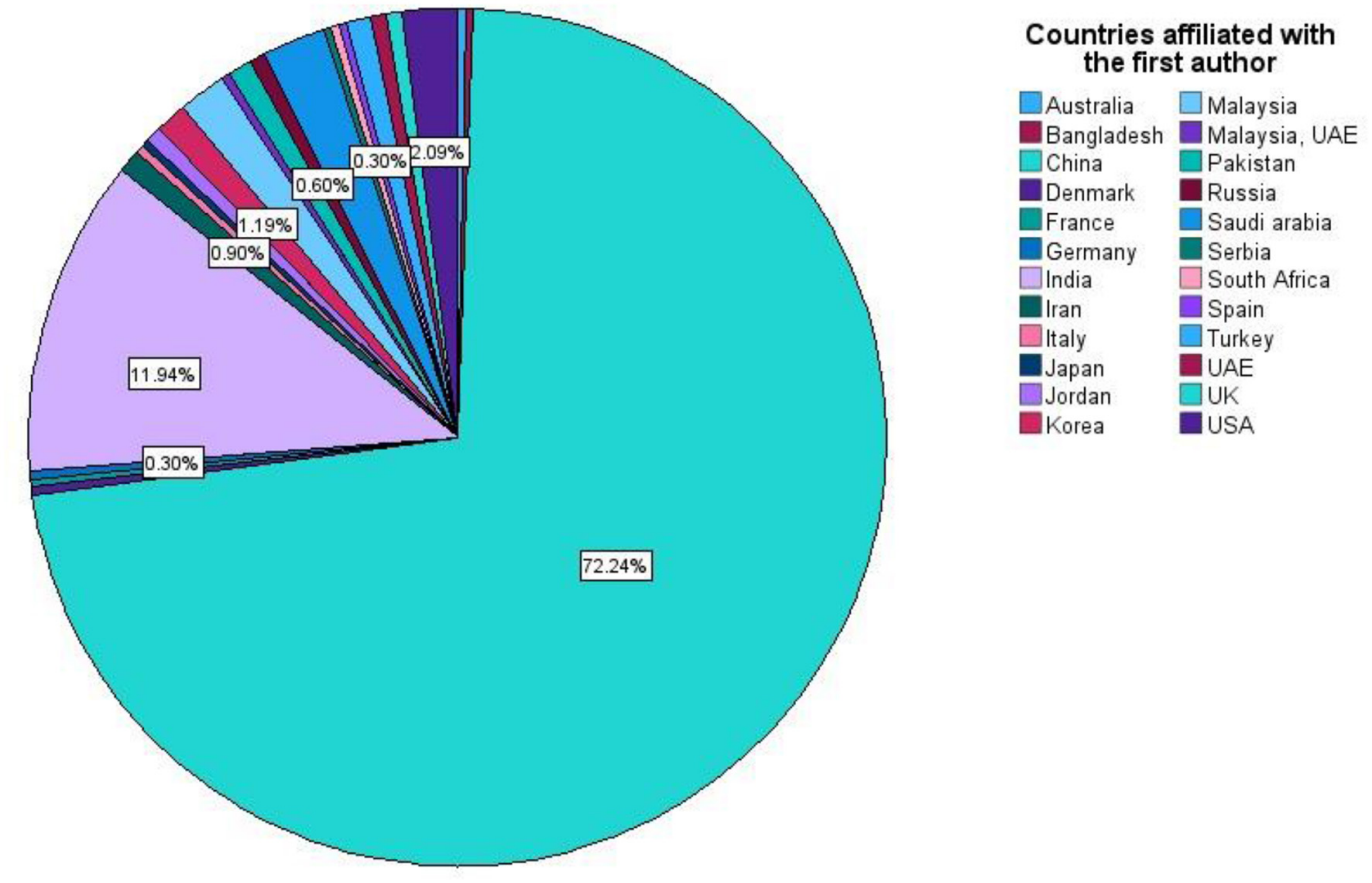
Countries affiliated with the first authors of retracted AI-related articles.

**Figure 7:**
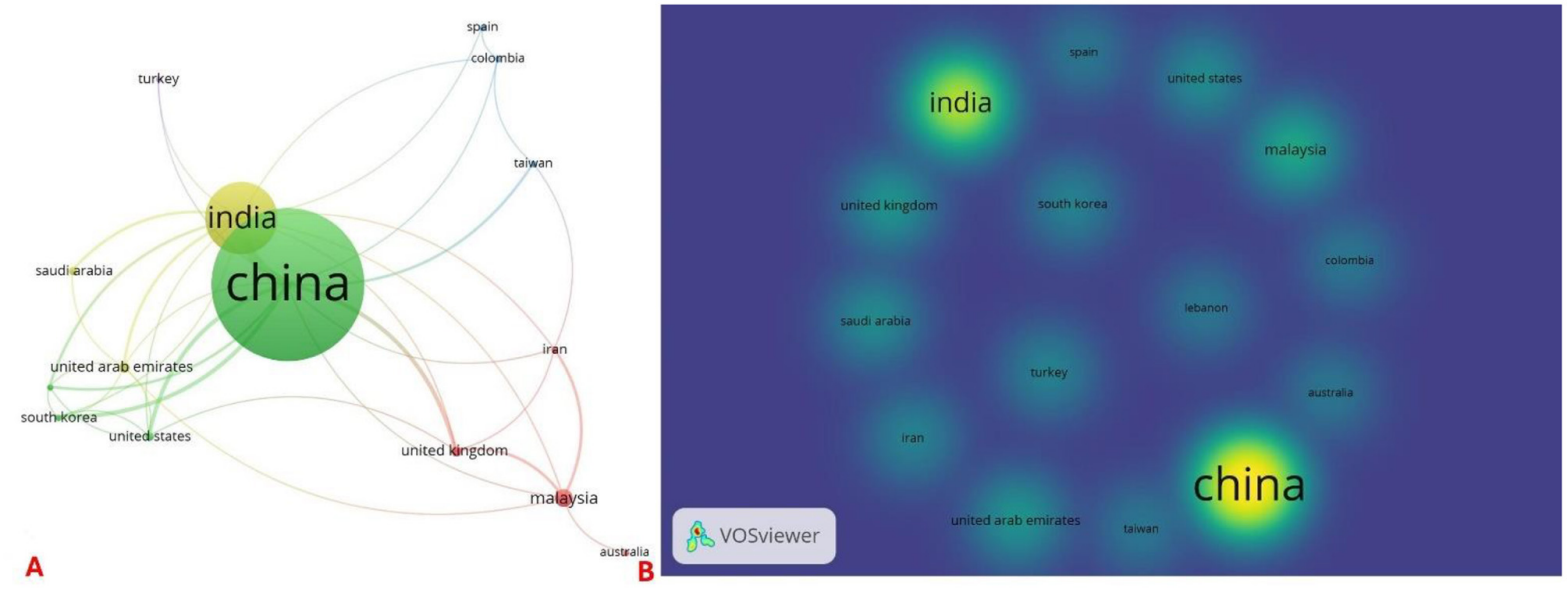
Country clusters of authors and country-wise density of citations included in the retracted AI-related articles. **(A)** Cluster of countries associated with authors of AI-related retracted articles; and **(B)** country-wise citation density plot of AI-related retracted articles.

Geographically, China accounted for 72.2% (242/335) of first authors, followed by India (40, 11.9%; Figure 6). Co-authorship analysis revealed four primary country clusters, with collaborations predominantly centered around China and India (Figure 7A). Citation density was also highest for China, followed by India (Figure 7B).

### Funding and conflict of interest

Regarding the funding details, 108 (32.2%) articles reported obtaining funding for their study, 29 (8.7%) had no funding and 193 (57.6%) did not report details, and 5(1.5%) retracted articles could not be assessed for this analysis. Of the articles reporting obtaining funding, 89 (82.4%) were from China, 4 (3.7%) from Saudi Arabia, 3 (2.8%) each from Malaysia and India, and one (0.9%) each from France, Japan, Korea, Pakistan, Spain, United Arab Emirates, and the USA.

Similarly, for conflict-of-interest statement, 263 (78.5%) reported absence of any conflict of interest, 68 (20.3%) did not report and 4 (1.2%) could not be assessed for this item analysis.

### Reasons for retraction of AI-related articles

As detailed in Table 1, compromised peer review emerged as the predominant reason for retraction among AI-related articles. A significant proportion of retractions (127, 37.8%) did not specify precise reasons, instead citing one or more generic concerns.

**Table 1:** Summary of reasons specified in the retracted AI-related articles.

**Specified reasons for retraction**	**Number (%)**
One or more of the following: • Compromised peer review • Discrepancies in journal's/special issue's scope • Inappropriate citations • Incoherent, meaningless and/or irrelevant content	85 (25.4)
Compromised peer review	78 (23.3)
One or more of the following: • Compromised peer review • Discrepancies in journal's/special issue's scope • Inappropriate citations • Incoherent, meaningless and/or irrelevant content • Non-compliance with human-subject research ethical reporting requirements	42 (12.5)
All the following: • Compromised editorial handling • Compromised peer review • Inappropriate citations • Discrepancies in journal's/special issue's scope	38 (11.3)
Plagiarism (text/image/results)	24 (7.2)
Compromised editorial handling and peer review process	18 (5.4)
Compromised editorial handling, peer review process and inappropriate citations	16 (4.8)
Compromised editorial handling and plagiarism	7 (2.1)
Data falsification	4 (1.2)
Copyright infringement	3 (0.9)
Compromised peer review and plagiarism	2 (0.6)
Compromised peer review and inappropriate citations	2 (0.6)
Authorship issues	2 (0.6)
Inappropriate citations	1 (0.3)
Conceded the use of AI in article preparation	1 (0.3)
Non-compliance with human-subject research ethical reporting requirements	1 (0.3)
Statistical errors	1 (0.3)
Concerns regarding the reliability and validity of the article	1 (0.3)
Article contained parts of unpublished manuscript	1 (0.3)
Erroneously republished by the journal	1 (0.3)
All the following: • Compromised editorial handling • Compromised peer review • Plagiarism	1 (0.3)
All the following: • Inappropriate citations • Conceded the use of AI in article preparation • Non-compliance with human-subject research ethical reporting requirements	1 (0.3)
All the following: • Inappropriate citations • Compromised peer review process • Incoherent, meaningless and/or irrelevant content • Discrepancies in journal's/special issue's scope	1 (0.3)
Compromised peer review process and data manipulation	1 (0.3)
Plagiarism and authorship issues	1 (0.3)
All the following: • Inappropriate citations • Image irregularities • Incoherent, meaningless and/or irrelevant content	1 (0.3)

AI, Artificial intelligence.

The retraction process was overwhelmingly initiated by journals, with editors or third-party audits responsible for 329 cases (98.2%). In contrast, authors independently requested retraction in only 5 instances (1.5%), while 1 case (0.3%) involved joint editor-author initiation. Statistical analysis revealed no significant difference in the time elapsed between publication and retraction when comparing journal-initiated vs. author-initiated cases (*p* = 0.2).

Among author-initiated retractions, plagiarism accounted for 2 cases (40%), with single instances (20% each) attributed to: ethical non-compliance, statistical errors, and inclusion of unpublished manuscript content. These author-led retractions originated from first authors in China, India, Germany, Japan, and the United States. Of these, three (60%) articles received funding, achieved a median citation count of 4.5, and appeared in journals with impact factors ranging from 0.2 to 10.7. Three articles (60%) were published in Q1 journals, all focused on therapeutics, and all represented original research published in regular issues, with a median retraction timeframe of 365 days (range: 68–691).

Author responses to retraction notices revealed substantial non-engagement, with 236 articles providing no response and 4 cases where contact attempts failed. Among the 96 cases with documented responses, authors failed to reply in 43 instances (44.8%), contested the retraction in 34 cases (35.4%), fully agreed in 15 cases (15.6%), and provided partial agreement in 4 cases (4.2%).

In cases where authors agreed to retraction, journal editors initiated the action in 10 instances (66.7%). The underlying reasons included plagiarism (5 cases, 33.3%), copyright infringement (3 cases, 20%), and compromised peer review (3 cases, 20%). Single cases (6.7% each) involved: ethical reporting violations, unpublished content inclusion, statistical errors, and combined issues of editorial mishandling, peer review deficiencies, citation irregularities, and scope discrepancies. The corresponding authors of these retracted works were primarily based in China (6 cases) and the United States (4 cases), with individual contributions from Japan, Germany, Denmark, Spain, and India. These articles demonstrated a median citation count of 5 (range: 1–53), with 7 (46.7%) receiving funding. The publishing journals were predominantly Q1 (7, 46.7%) and Q2 (6, 40%), with all but one appearing in regular issues.

### Keywords and thematic analysis

**Figure 8:**
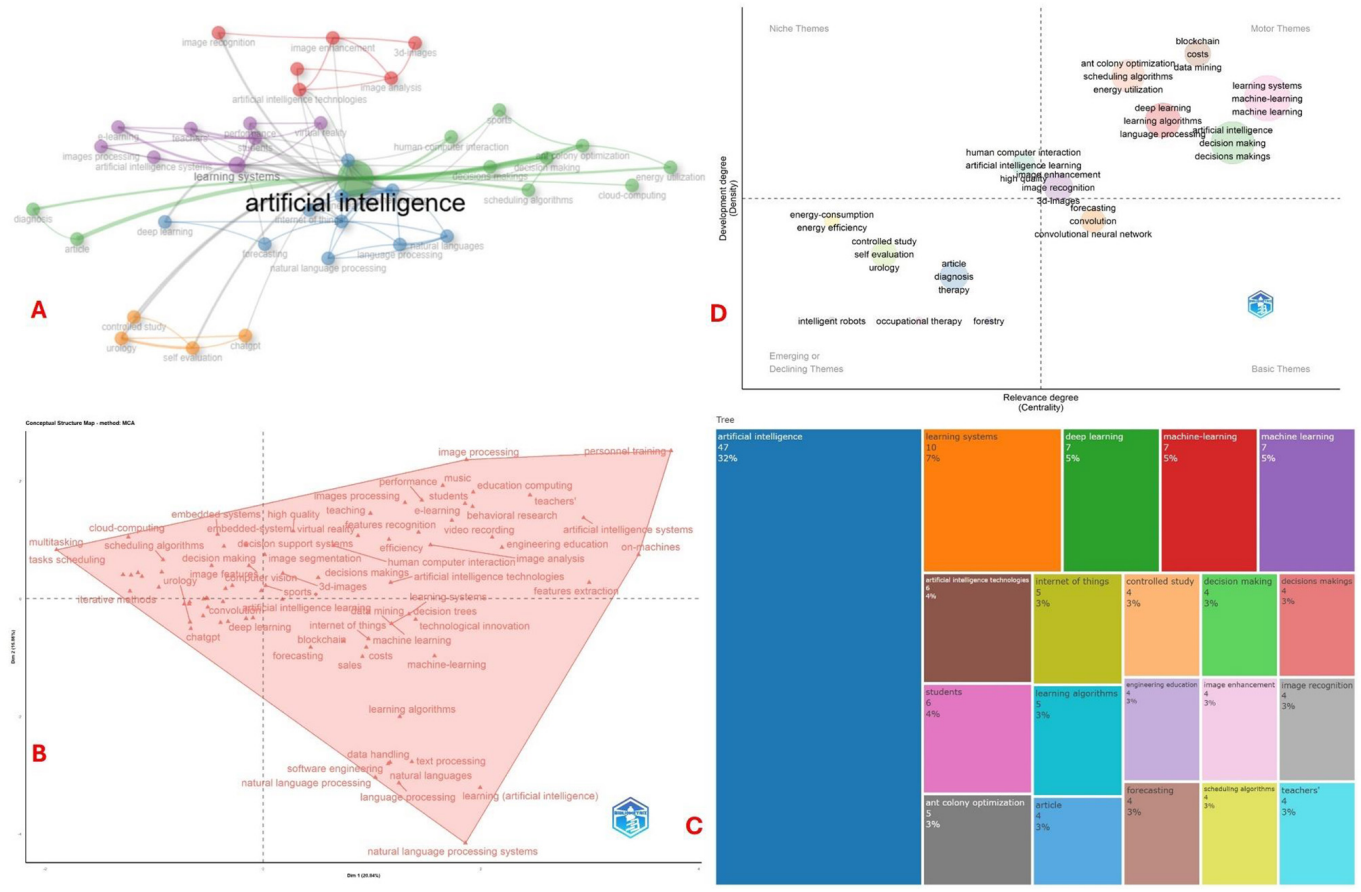
Analyses of author keywords. **(A)** Cluster of author keywords; **(B)** multidimensional factorial analysis; **(C)** treemap of author keywords; and **(D)** thematic map. The upper left panel presents a keyword co-occurrence network generated using author keywords, where node size reflects the frequency of each keyword, node color indicates cluster membership based on co-occurrence patterns, and edge thickness corresponds to the strength of co-occurrence links. “Artificial intelligence” emerged as the dominant and most central term, forming multiple interconnected clusters related to learning systems, deep learning, machine learning, image analysis, natural language processing, decision-making algorithms, and human–computer interaction. The lower right panel displays the thematic map of the most frequent author keywords, with each rectangle sized according to keyword occurrence within the dataset. Consistent with the network map, “artificial intelligence” accounted for the largest share, followed by thematic areas such as learning systems, deep learning, machine learning, artificial intelligence technologies, and domain-specific applications including Internet of Things, decision-making, image enhancement, and scheduling algorithms. Together, these visualizations highlight the conceptual focus and thematic distribution of research within the analyzed corpus.

Author keyword analysis revealed five thematic clusters, with “artificial intelligence” being the most frequent, followed by domains like education, natural language processing, image analysis, clinical evaluation, and decision-making algorithms (Figure 8A). The multiple correspondence analysis (Figure 8B) displays two dimensions, Dim 1 (20.84%) and Dim 2 (16.65%), capturing the variance in the dataset and organizing keywords based on co-occurrence patterns. The map reveals a wide spread of thematic areas, with clearly distinguishable conceptual groupings. In the upper right quadrant, terms such as “image processing,” “personnel training,” “education computing,” and “artificial intelligence systems” suggest a strong linkage between AI applications in education and image analysis technologies. Toward the bottom center and right, a dense cluster centers around “natural language processing,” “text processing,” and “software engineering,” reflecting a focus on computational linguistics and system-based learning algorithms. The central cluster, encompassing terms like “machine learning,” “data mining,” and “decision trees,” appears to be a core theme across the dataset. Notably, the lower left quadrant includes keywords like “chatgpt,” “deep learning,” and “blockchain,” which may represent more contemporary and emerging topics in AI research that have also experienced retractions. The relatively peripheral placement of terms like “urology” and “sports” indicates their more niche or interdisciplinary application.

The treemap (Figure 8C) visualization illustrates the frequency and relative prominence of key terms associated with retracted articles on artificial intelligence. “Artificial intelligence” overwhelmingly dominates the landscape, appearing in 47 instances and accounting for 32% of the total keywords, confirming its centrality in the dataset. Secondary yet significant themes include “learning systems” (10 occurrences, 7%) and a cluster of interrelated terms such as “deep learning,” “machine-learning,” and “machine learning,” each contributing 5%, suggesting that various machine learning paradigms are frequently represented in the retracted literature. Other notable terms like “artificial intelligence technologies,” “students,” “internet of things,” and “learning algorithms” highlight a broad range of AI applications and stakeholders. Several terms, including “controlled study,” “decision making,” “engineering education,” “image enhancement,” and “scheduling algorithms,” each contribute 3%, indicating their moderate yet consistent appearance.

The thematic map (Figure 8D) classifies themes from retracted AI-related publications based on two dimensions: centrality (relevance degree) and density (development degree). In the upper right quadrant, designated as “Motor Themes,” highly developed and central topics such as “machine learning,” “learning systems,” “decision making,” “deep learning,” and “language processing” are present, indicating that these areas are both pivotal to the AI domain and well-developed in the context of retracted literature. In contrast, the lower left quadrant, labeled “Emerging or Declining Themes,” includes topics like “intelligent robots,” “occupational therapy,” and “forestry,” suggesting limited development and marginal relevance, these could either represent emerging frontiers or areas of waning interest. The upper left quadrant, representing “Niche Themes,” is remarkably sparse, indicating few specialized but well-developed subtopics in the retracted literature. Meanwhile, the lower right quadrant, categorized as “Basic Themes,” includes foundational yet less developed topics such as “convolutional neural network,” “forecasting,” and “3D images,” which are relevant but may lack conceptual maturity or interdisciplinary integration.

## Discussion

### Statement of key findings

Our bibliometric analysis of 335 retracted AI-related articles reveals several critical findings that merit discussion. The study identified a striking predominance of retractions in engineering disciplines (30.4%), with China accounting for nearly three-quarters (72.2%) of first authors. The temporal analysis showed a dramatic surge in retractions during 2023 (46.3% of cases), following peak publication rates in 2022. Remarkably, compromised peer review emerged as the most frequently cited reason for retraction, though 37.8% of cases lacked specific justification. The data demonstrate concerning publication patterns, with special issue articles showing significantly accelerated peer review timelines (*p* = 0.016) and the median time-to-retraction exceeding 18 months (550 days). Journal-initiated retractions dominated (98.5%), while author responses revealed substantial resistance, with 35.4% of contacted authors disputing the retraction decision. The citation impact analysis showed that 51.1% of retracted articles maintained above-average field citation ratios (>1), suggesting their continued influence post-retraction. These findings collectively highlight systemic vulnerabilities in AI-related research publication, particularly concerning peer review integrity, international publishing practices, and the persistence of problematic literature in the scientific record.

### Comparison with existing literature

The findings of this bibliometric analysis provide critical insights into the landscape of retracted AI-related research, revealing systemic challenges that warrant urgent attention from the scientific community. Our study, encompassing 335 retracted articles, uncovers concerning trends in publication ethics, peer review integrity, and the persistence of flawed research in the scholarly record. We have contextualized the findings of this study within the broader framework of academic publishing, examines their implications for research integrity, and proposes actionable recommendations to mitigate these issues.

### Temporal, geographical, and disciplinary patterns in retractions

Our analysis reveals interconnected trends in the timing, origin, and focus of AI-related retractions. A dramatic surge occurred in 2023, with 46.3% of retractions following peak publication rates in 2022. This lag, reflected in a median retraction timeframe of 550 days, suggests a critical delay in misconduct detection, allowing problematic articles to circulate and potentially mislead the field (Chauvin et al., 2019; Panahi and Soleimanpour, 2023). This issue is exacerbated in fast-moving AI research and is particularly pronounced for articles in special issues, which had significantly accelerated peer review timelines (*p* = 0.016), hinting at expedited but less rigorous evaluation processes.

Geographically, retractions were heavily concentrated, with China accounting for 72.2% of first authors, a pattern consistent with analyses of retractions for randomly generated content (Lei et al., 2024) and systematic reviews (Shahraki-Mohammadi et al., 2024). This overwhelming predominance, alongside high retraction rates in other Asian countries (Oransky, 2018), points to systemic pressures within high-volume publishing systems. Factors may include strong institutional incentives tied to publication metrics, a “publish or perish” culture, and potential deficits in ethical oversight, which can distort academic priorities (Tian et al., 2016; Horta and Li, 2022).

These pressures intersect with specific disciplinary vulnerabilities. Retractions were concentrated in engineering (30.4%) and education (12.2%), fields characterized by rapid innovation cycles and emerging adoption of AI tools, respectively. This suggests environments where the drive for speed and novelty may sometimes come at the expense of methodological rigor, facilitating the publication and subsequent retraction of flawed AI research.

### Peer review compromises and retraction rationales

Compromised peer review was the most frequently cited reason for retraction, aligning with growing concerns about the manipulation of scholarly evaluation processes. The prevalence of this issue underscores how AI's potential to generate plausible but unverified content exacerbates existing weaknesses in peer review. The rise of paper mills and fraudulent peer review rings has been well-documented in biomedical publishing, but our findings suggest these practices are now infiltrating AI research. A manipulated peer review or fake peer review usually suggests misusing the automated submission system by authors in suggesting details of the non-existent reviewers by authors. However, in the present study, it has also been observed that compromised peer review processing has also been observed contributed by the editors. A comprehensive review of the retracted articles in the Retraction Watch database through February 2019 revealed that fabricated peer review emerged as the predominant cause of article retractions (Rivera and da Teixeira Silva, 2021). Contrastingly, analysis of reasons for retraction from authors of Indian institutions revealed that plagiarism accounted for 27% of cases, while data manipulation (falsification/fabrication) represented 26%, duplicate submissions made up 21% of retractions, incorrect data (12%), authorship disputes (4%), fraudulent peer review (3%), and violations involving research ethics or funding disclosures (2%; Sharma et al., 2023). Hence, regional and journal differences in the cause for retraction may exist and need to be explored in future studies. The fact that 98.5% of retractions were journal-initiated, with only 1.5% stemming from author self-correction, further emphasizes the reactive rather than proactive nature of current integrity measures.

### Author responses and the challenge of accountability

The resistance to retraction among authors, 35.4% disputed the decision, while 44.8% did not respond, reflects broader tensions in academic accountability. The high rate of non-response may indicate disengagement, difficulty in contacting authors, or a lack of clear retraction protocols. Articles where authors agreed to retractions were often linked to unambiguous violations (such as plagiarism and copyright infringement), whereas contested cases may involve disputes over subjective judgments or AI-related ambiguities. The lack of standardized criteria for AI-related misconduct complicates these decisions, as current retraction guidelines were not designed to address AI-specific infractions. Also, we observed that the author-initiated retractions in the present study were only 1.5% of the retracted articles. A recent investigation examined perspectives on research integrity through two approaches: in-depth interviews with six international funding agency representatives and a subsequent survey of 224 US-based grant reviewers (Ribeiro et al., 2023). These participants had evaluation experience with major funding bodies including the National Science Foundation and National Institutes of Health. The findings revealed their attitudes regarding how literature corrections and article retractions impact funding decisions. Their analysis demonstrated that most respondents viewed the process of amending published research, whether due to genuine mistakes or ethical violations, as a crucial safeguard for maintaining scientific credibility within the grant allocation system and history of retractions and self-corrections were not observed to influence the grant review (Ribeiro et al., 2023).

### Impact of retracted research and citation persistence

Perhaps the most alarming finding is that 51.1% of retracted articles-maintained field citation ratios >1, indicating their continued influence post-retraction. This persistence mirrors patterns seen in other fields, where retracted studies are often cited without acknowledgment of their retracted status. The median citation counts of five (with some articles receiving up to 96 citations) demonstrates how retracted AI research continues to shape scholarly discourse. Research has demonstrated that retracted articles continue to be referenced in scholarly work despite ethical prohibitions against such citations (Bolland et al., 2022; Fanelli et al., 2022). Multiple investigations have identified several systemic factors contributing to this persistent citation problem (Schmidt, 2024). These include instances where retractions are not clearly marked, difficulties in locating retraction announcements, inadequate database indexing of withdrawn publications, delays in issuing retractions, insufficient disclosure about why articles were retracted, and inconsistent consequences for authors of retracted work (Schmidt, 2024). These challenges collectively create an environment where problematic publications remain in circulation and continue to influence subsequent research. Additionally, retracted articles were observed to be highly population with altmetric score as high as 240 as observed in this study. A recent study that assessed the media and social attention of the retracted articles revealed a random analysis of 572 retracted PubMed articles compared to matched unretracted controls revealed that 56.1% (*n* = 1,687) attracted some altmetric attention, with 5.5% (*n* = 165) being highly popular (almetric score>20; Serghiou et al., 2021). Also, 1% (31/2,953) of the retracted articles accumulated over 100 citations. Compared to controls, retracted articles garnered greater Altmetric attention (23/31 groups; *p* = 0.01), demonstrating their disproportionate visibility persists post-retraction (Serghiou et al., 2021).

### Structural vulnerabilities in academic publishing

Our analysis revealed several structural weaknesses that facilitated the propagation of flawed AI research. First, the predominance of retractions in Q2 journals (59.7%), traditionally viewed as mid-tier but credible, suggests that even well-established outlets struggle with AI-related misconduct detection. Second, the median impact factor of journals with retracted AI-related articles was observed to be 2. Previous research (Wiedermann, 2016) demonstrated that most retractions in intensive care medicine occurred in reputable journals (*n* = 2,444 articles). This suggests that high-quality journals possess more effective mechanisms for detecting research misconduct and scientific inaccuracies (Fang and Casadevall, 2011). However, the prestige associated with publishing in such journals, which enhances researchers' academic standing (Smith, 2008; Szklo, 2008; Fersht, 2009), may paradoxically increase the likelihood of fraudulent practices occurring in these venues (Fang and Casadevall, 2011). Third, the high proportion of special issue retractions (82.2%) with accelerated review timelines points to a dangerous trade-off between speed and rigor, particularly in emerging fields where guest editors may lack oversight.

### Toward solutions: recommendations for stakeholders

The key recommendations to various stakeholders emerging from the key findings of the present study are outlined in Table 2. Addressing these challenges requires coordinated action across the academic ecosystem. Journals must adopt stricter AI-use disclosure policies, including detailed reporting of AI's role in writing, data analysis, and figure generation. Funding agencies and institutions should reevaluate incentive structures that prioritize quantity over quality, particularly in high-volume research systems. Publishers could implement AI-driven screening tools to flag suspicious manuscripts, such as those with boilerplate text or inconsistent methodological descriptions. Database providers like Scopus and Web of Science must improve retraction alert systems, ensuring that citation tracking clearly flags withdrawn articles. Finally, the development of field-specific guidelines for AI-related misconduct, distinguishing between intentional deception and unintentional misuse, would help standardize retraction decisions.

**Table 2:** Evidence-based recommendations for stakeholders to mitigate AI-related research misconduct and strengthen publication integrity.

**Stakeholder group**	**Recommendations**	**Rationale/supporting evidence from study**
Journals and publishers	• Strengthen oversight of special issues. Standardize and audit peer review processes for special issues to prevent expedited but compromised review. • Develop and apply field-specific guidelines for AI-related misconduct. Clearly define and distinguish between intentional deception and unintentional misuse of AI tools to standardize retraction decisions. • Enhance transparency in retraction notices. Move beyond generic reasons and provide specific, actionable explanations for the retraction to educate the community.	• Compromised peer review was the top reason for retraction. • 37.8% of retraction notices lacked precise justifications. • Special issue articles had significantly accelerated peer review timelines (*p* = 0.016).
Researchers and institutions	• Prioritize rigorous methodology and human oversight over publication speed. • Avoid the “publish or perish” pressure that may lead to reliance on unverified AI outputs. • Foster a culture of ethical AI use and self-correction. Encourage author-initiated retractions for honest errors. • Provide training on the ethical use of AI in research. Focus on its limitations, risks of generating misleading content/inappropriate citations, and disclosure requirements.	• Median time from submission to acceptance was only 47.5 days, suggesting rapid turnover. • 72.2% of first authors were from China, pointing to systemic pressures in high-volume publishing systems. • Only 1.5% of retractions were author-initiated.
Bibliographic database providers	• Improve the visibility and linking of retraction status. Ensure retraction alerts are prominent and propagate across all journal and citation interfaces. • Develop automated alerts for citing retracted work. Notify authors when they reference a retracted article to curb its persistent influence.	• 51.1% of retracted articles had an above-average Field Citation Ratio (>1), indicating continued citation post-retraction.
Funding agencies & academic societies	• Re-evaluate research assessment metrics. Move beyond quantitative publication counts and journal prestige (Q1/Q2) toward quality and integrity indicators. • Support the development of AI-driven screening tools. Fund initiatives to create tools that help journals detect AI-generated text, image irregularities, and citation manipulation. • Establish clear consequences for misconduct. Develop standardized sanctions for authors and institutions involved in AI-related publication violations.	• Most retractions occurred in Q2 (59.7%) and Q1 (28.1%) journals. • 32.2% retracted articles reported obtaining funding. • Issues like inappropriate citations and image irregularities were identified causes.

### Strengths, limitations and future directions

This study offers several notable strengths that enhance its contribution to the literature on research integrity and AI-related publications. The comprehensive bibliometric approach, analyzing 335 retracted articles across multiple dimensions including temporal trends, disciplinary distribution, citation impact, and retraction characteristics, provides a robust empirical foundation for understanding the phenomenon. The inclusion of multiple analytical metrics, from traditional citation counts to modern altmetrics and field-normalized citation ratios, allows for a nuanced assessment of the ongoing influence of retracted AI research. By examining both the quantitative aspects (such as time-to-retraction and journal quartiles) and qualitative factors (including retraction reasons and author responses), the study bridges an important gap between bibliometric analysis and research ethics. The focus on AI-related retractions is particularly timely given the rapid adoption of these technologies in scholarly work, offering insights that are immediately relevant to publishers, institutions, and policymakers. Furthermore, the identification of specific vulnerabilities in special issue publications and geographical concentrations of retractions provides actionable intelligence for targeted interventions. To ensure the robustness and accuracy of our dataset, a multi-step validation protocol was implemented. First, following the initial export from Scopus, all 1,152 records were programmatically and manually checked for duplicates based on digital object identifier, title, and Scopus identifier, resulting in the removal of 51 entries. Second, to verify the retraction status of each included article, we performed a cross-check by directly accessing the official retraction notice on the publisher's website or within the journal platform for all the final 335 articles. This confirmed that the Scopus retraction flag was accurate in all sampled cases. Third, to ensure the consistency of data extraction, key variables, including publication/retraction dates, document type, retraction reason, and citation counts, were independently extracted by two authors for the entire dataset. Finally, logical validation was performed on the compiled dataset to identify and rectify any inconsistencies, such as retraction dates preceding publication dates or mismatched journal metrics. The study's rigorous methodology, employing multiple validated bibliometric tools and statistical analyses, ensures the reliability of its findings while maintaining transparency through clear reporting of all data parameters. These strengths collectively position this work as a significant reference point for future research on publication ethics in the age of artificial intelligence.

Our study has certain limitations that should be considered when interpreting the findings. First, the search strategy was confined to the Scopus database. While Scopus is one of the largest and most rigorously curated multidisciplinary bibliographic databases, with strong capabilities for identifying retracted publications, the exclusion of other databases like Web of Science, PubMed, or specialized registries like Retraction Watch may mean some eligible retracted articles were not captured. This single-source approach could introduce a selection bias, potentially affecting the comprehensiveness of our dataset, particularly for articles in journals not indexed by Scopus. However, Scopus's extensive coverage and its integration with the bibliometric analysis tools required for this study provided a robust and consistent foundational dataset. Future research could employ a multi-database search strategy to further validate and expand upon these findings. Second, our analysis relied on the retraction reasons formally provided in the database or publisher notices; we did not conduct independent investigations into the veracity of these stated causes, which may sometimes be incomplete or vague. Further, the decision on not searching Retraction Watch database was primarily methodological, to ensure a consistent and reproducible dataset extracted from a single, structured source (Scopus) that includes formal retraction notices and allows for standardized bibliometric export. While Retraction Watch is a valuable resource for monitoring retractions, its data structure is not directly compatible with the automated bibliometric software workflows required for our analysis. As a bibliometric analysis of secondary data, this study is subject to several inherent biases and limitations related to data sourcing and interpretation. First, while our screening process followed predefined criteria, screening bias may exist as decisions regarding the relevance of an article to “AI-related” research involved a degree of subjective judgment, particularly for articles where AI was a peripheral component. Second, our reliance on a single database (Scopus) introduces potential database indexing inconsistencies; the completeness and timing of retraction flagging can vary across platforms, and some relevant retractions in non-indexed journals may have been missed. Third, a significant source of variability stems from retraction notice heterogeneity. Notices often lack standardized terminology or detailed explanations, with a substantial proportion (37.8% in our dataset) citing generic concerns rather than specific violations. This variability complicates the categorical analysis of retraction causes. Finally, our study involved the manual interpretation and categorization of retraction rationales. This process, while conducted systematically, requires translating often vague or composite publisher statements into discrete categories, introducing a potential for interpretive bias. We mitigated this by having two independent reviewers perform the categorization with high inter-rater agreement, followed by consensus resolution for discrepancies. Acknowledging these potential biases is essential for contextualizing our results, which should be interpreted as reflecting the patterns present within the structured scholarly record rather than as a fully exhaustive or unequivocal account of all AI-related misconduct. Additionally, the evolving nature of AI tools means that newer forms of misconduct (e.g., deepfake data, AI-generated images) may not yet be reflected in retraction data. Future research should expand to include preprints and conference proceedings, where AI-related errors may first appear. Longitudinal tracking of retraction trends could also clarify whether current measures are effectively curbing misconduct.

## Conclusion

The retraction of AI-related research is not merely a metric of failure but a critical indicator of systemic stresses in modern academia. Our findings highlight how the intersection of AI's disruptive potential and existing publishing vulnerabilities creates fertile ground for misconduct. While AI offers transformative opportunities for science, its misuse threatens to erode trust in scholarly communication. Proactive measures, ranging from technological safeguards to cultural shifts in research evaluation, are urgently needed to ensure that the AI revolution in academia strengthens, rather than undermines, scientific integrity. The time to act is now, before the deluge of AI-assisted research overwhelms the systems designed to guard its validity.

## Data Availability

The datasets used in this study were obtained with the search strategy provided in the methods section of this manuscript in Scopus database.
