# Supplementary material for: Artificial intelligence in the retraction spotlight: trends, causes and consequences of withdrawn AI literature through a systematic bibliometric review
Source: Front Res Metr Anal. 2026 Jan 20;10:1737168. doi: 10.3389/frma.2025.1737168 (PMC12864414; doi:10.3389/frma.2025.1737168)
Supplement: Supplementary file 1 [file Data_Sheet_1.docx]

**Electronic Supplementary Figure 1. Top 10 journals reported with retractions of AI-related articles in the top journals.**

**
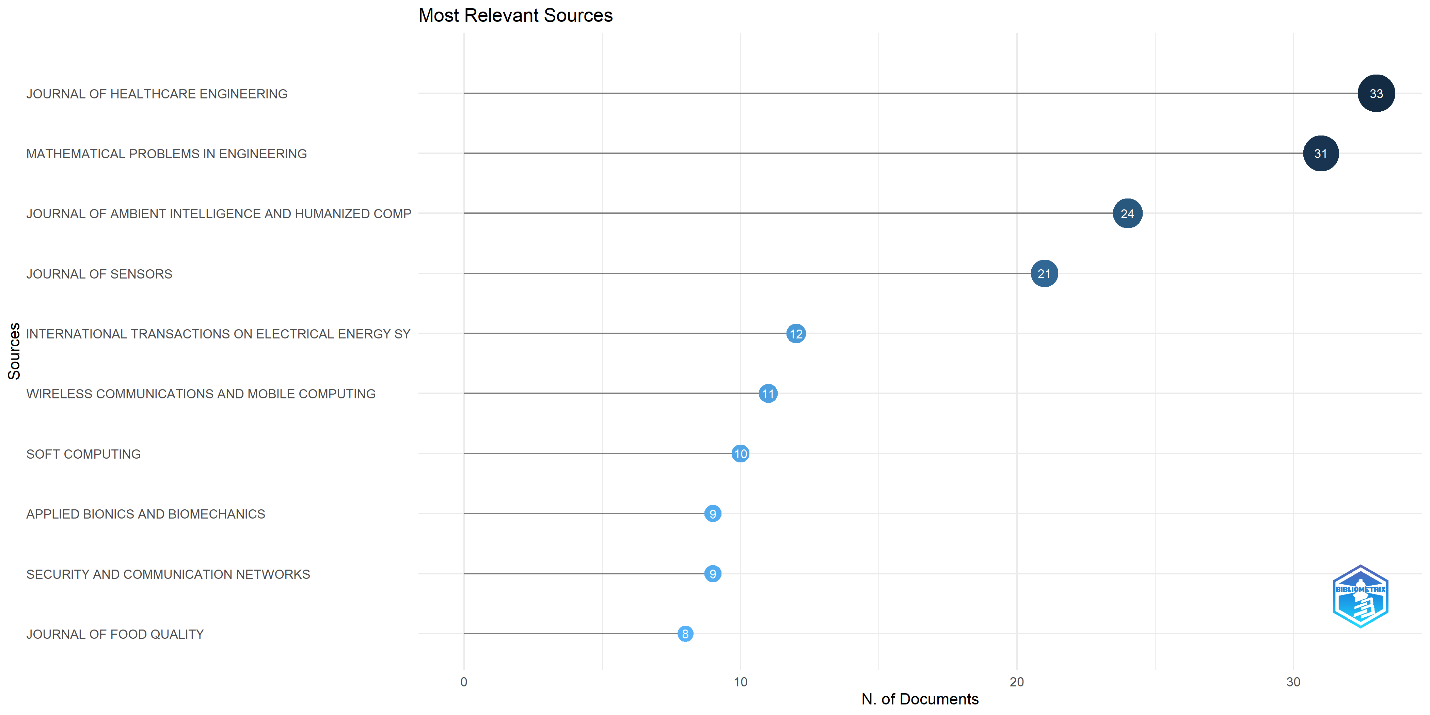
**

**Electronic Supplementary Figure 2. Top 10 journals with top H-index (by local impact) identified with retractions of AI-related articles.**

**
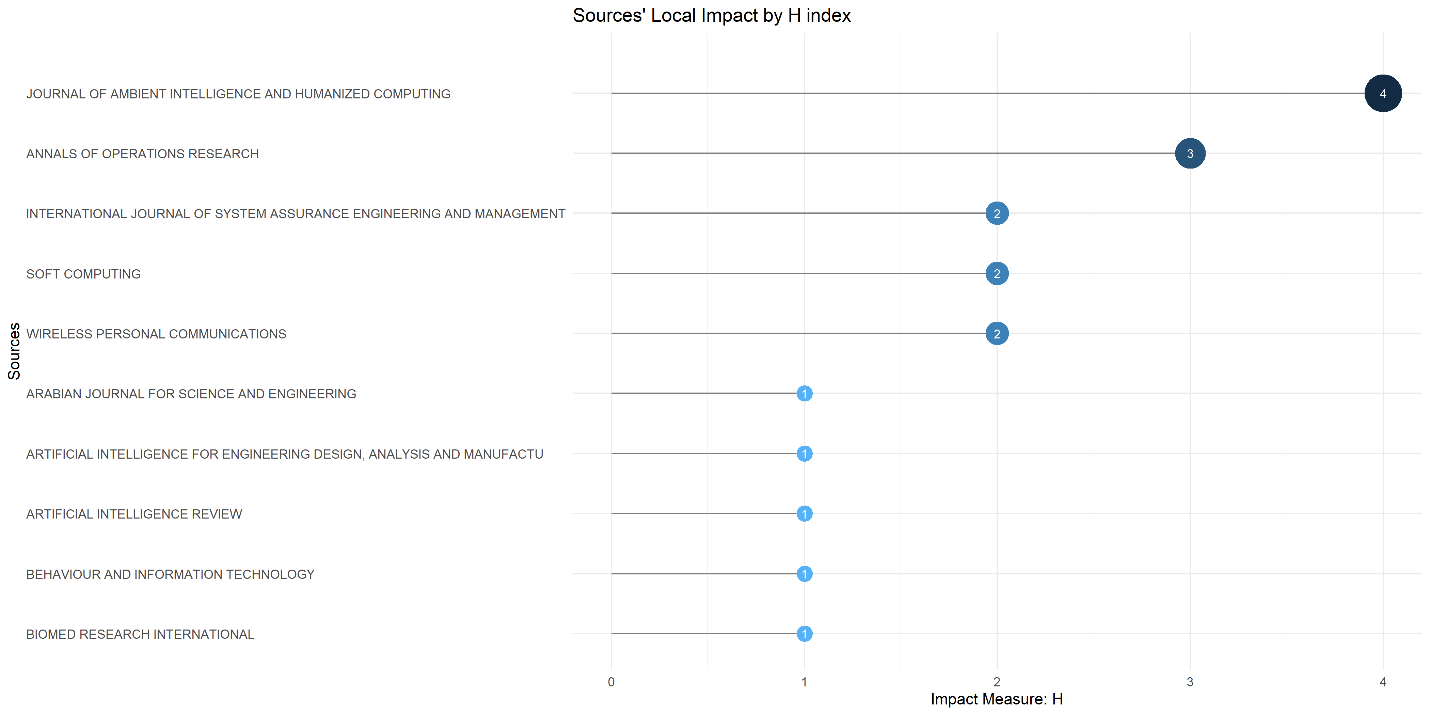
**
